# Diacylglycerol Kinase alpha is Involved in the Vitamin E-Induced Amelioration of Diabetic Nephropathy in Mice

**DOI:** 10.1038/s41598-017-02354-3

**Published:** 2017-06-01

**Authors:** Daiki Hayashi, Keiko Yagi, Chihong Song, Shuji Ueda, Minoru Yamanoue, Matthew Topham, Toshinobu Suzaki, Naoaki Saito, Noriaki Emoto, Yasuhito Shirai

**Affiliations:** 1 Department of Applied Chemistry in Bioscience, Graduate School of Agricultural Science, Faculty of Agriculture, Kobe University, Kobe, Japan; 2 Department of Clinical Pharmacy, Kobe Pharmaceutical University, Kobe, Japan; 3 Department of Biology, Graduate School of Science, Faculty of Science, Kobe University, Kobe, Japan; 4 Huntsman Cancer Institute, University of Utah, Salt Lake City, UT USA; 5 Laboratory of Molecular Pharmacology, Biosignal Research Center, Kobe University, Kobe, Japan

## Abstract

Diabetic nephropathy (DN) is one of vascular complications of diabetes and is caused by abnormal protein kinase C activation as a result of increased diacylglycerol (DG) production in diabetic hyperglycaemia. Diacylglycerol kinase (DGK) converts DG into phosphatidic acid. Therefore, it is expected that the activation of DGK would ameliorate DN. Indeed, it has been reported that vitamin E (VtE) ameliorates DN in rat by activating DGK, and we recently reported that VtE specifically activates DGKα isoform *in vitro*. However, whether DGKα is involved in the VtE-induced amelioration of DN *in vivo* remains unknown. Therefore, we investigated the VtE-induced amelioration of DN in wild-type (DGKα^+/+^) and DGKα–deficient (DGKα^−/−^) mice in which diabetes was induced by streptozocin. Several symptoms of DN were ameliorated by VtE treatment in the DGKα^+/+^ mice but not in the DGKα^−/−^ mice. Moreover, transmission electron microscopy of glomeruli and immunofluorescent staining of glomerular epithelial cells (podocytes) indicated that VtE ameliorates podocyte pathology and prevents podocyte loss in the DGKα^+/+^ mice but not in the DGKα^−/−^ mice. We showed that VtE can ameliorate DN in mice and that DGKα is involved in the VtE-induced amelioration of DN *in vivo*, suggesting that DGKα is an attractive therapeutic target for DN.

## Introduction

Diabetic nephropathy (DN) is one of multiple serious vascular complications of diabetes, and causes albuminuria and failure of glomerular filtration. DN is a disorder of the microvasculature that is caused by sustained hyperglycaemia; hyperglycaemia results in the production of diacylglycerol (DG) from glucose by *de novo* synthesis^1, 2^, and the DG produced by this pathway abnormally activates protein kinase C (PKC)^3, 4^. This abnormal activation of PKC is one of the causes of DN, but many other factors also contribute^5, 6^. Indeed, enhancement of the DG-PKC pathway has been reported in the vascular tissues of diabetic rats^7, 8^. Together, these data suggest that normalisation of the DG-PKC pathway in glomeruli is important to ameliorate DN.

Diacylglycerol kinase (DGK) is an enzyme that converts DG into phosphatidic acid (PA), which activates phosphatidylinositol 4-phosphate 5-kinase^9^ and mammalian target of rapamycin (mTOR)^10^. By contrast, DGK can attenuate PKC activity by reducing the amount of DG, which suggests that DGK can normalise abnormal PKC activity during hyperglycaemia. Indeed, it was reported that d-α-tocopherol (vitamin E; VtE) ameliorates DN in diabetic rats by normalising the DG-PKC pathway through DGK activation^11^. To date, 10 subtypes of mammalian DGK have been reported^12–14^. We previously showed that VtE specifically translocated DGK alpha (DGKα) from the cytoplasm to the plasma membrane which is a hallmark of its activation^15^. Indeed, VtE increased activity of DGKα^15^. These results suggest that DGKα is involved in the VtE-induced amelioration of DN. However, it remains unknown whether DGKα is involved in this process *in vivo* because the previous study was performed *in vitro*. Therefore, this study investigated the involvement of DGKα in the VtE-induced amelioration of DN in DGKα-deficient (DGKα^−/−^) mice in which diabetes had been induced by streptozocin (STZ).

## Results

### The effects of VtE on blood glucose levels and body weight

We first compared the phenotype of DGKα^+/+^ and DGKα^−/−^ mice before STZ treatment. There was no significant difference in body weight (DGKα^+/+^: 19.81 ± 1.34 g, DGKα^−/−^: 20.11 ± 1.98 g), and blood glucose levels were similar between DGKα^+/+^ and DGKα^−/−^ mice. However, the blood glucose levels of the DGKα^−/−^ mice were slightly higher than those of the DGKα^+/+^ mice (DGKα^+/+^: 159.53 ± 21.19 mg/dL, DGKα^−/−^: 165.89 ± 31.83 mg/dL). In addition, the levels of blood biochemical markers, including LDH (indicative of liver function), AMY (indicative of pancreatic function), T-CHO, TG and NEFA (indicative of lipid metabolism) were similar between groups.

**Figure 1:**
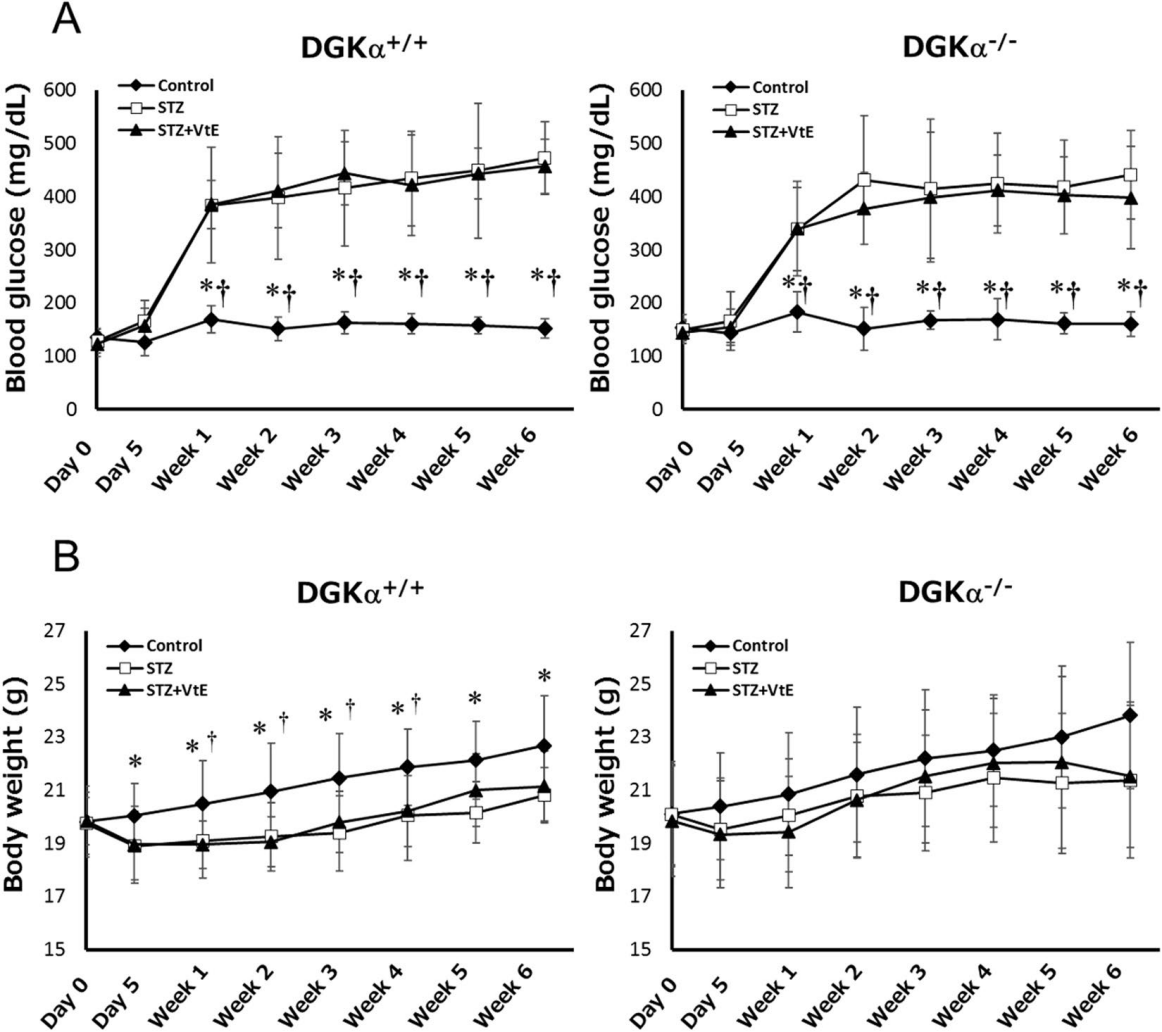
Changes in blood glucose levels and body weight. The blood glucose levels (**A**) and body weight (**B**) of the mice in each group were measured before and after STZ administration (day 0 and day 5), and every week thereafter until 6 weeks. Number of the mice in each group: DGKα^+/+^ Control: n = 12~18, STZ: n = 13~20, STZ + VtE: n = 8~11, DGKα^−/−^ Control: n = 12~18, STZ: n = 12~19, STZ + VtE: n = 9~11. *P < 0.01 vs. STZ; ^†^P < 0.01 vs. STZ + VtE.

After the final STZ administration, the blood glucose levels of the STZ-treated DGKα^+/+^ and DGKα^−/−^ mice increased at 1 week, confirming the induction of diabetes (DGKα^+/+^ STZ: 384 ± 109.19 mg/dL, DGKα^+/+^ STZ + VtE: 385.27 ± 45.78 mg/dL, DGKα^−/−^ STZ: 339.68 ± 89.63 mg/dL, DGKα^−/−^ STZ + VtE: 339 ± 77.8 mg/dL). The STZ-treated mice showed sustained high blood glucose levels until 6 weeks after the final STZ treatment (Fig. 1A). There was no significant difference in the blood glucose levels of the STZ-treated DGKα^+/+^ and DGKα^−/−^ mice (Fig. 1A); the mean blood glucose level during weeks 1–6 for the STZ-treated DGKα^+/+^ mice was 419.5 ± 106.53 mg/dL, and that for the STZ-treated DGKα^−/−^ mice was 408.03 ± 108.88 mg/dL. Furthermore, STZ treatment decreased the body weight of DGKα^+/+^ and DGKα^−/−^ mice (Fig. 1B). VtE treatment did not affect the blood glucose levels of the STZ-treated DGKα^+/+^ or DGKα^−/−^ mice (DGKα^+/+^: 424.68 ± 66.73 mg/dL, DGKα^−/−^: 386.98 ± 86.11 mg/dL), or body weight loss in the DGKα^+/+^ or DGKα^−/−^ mice (Fig. 1B), which is similar to the results that have been obtained in rats^11^.

Recently, we reported that DGKα is also involved in insulin secretion in pancreatic β-cells^16^. Indeed, the blood glucose concentrations of DGKα^−/−^ mice were slightly higher than those of the DGKα^+/+^ mice (Fig. 1A). However, the blood glucose concentrations of the DGKα^+/+^ and DGKα^−/−^ mice with STZ-induced diabetes were not significantly different, because STZ is toxic to pancreatic β-cells. In other words, the effect of DGKα loss was ignorable in this study because insulin secretion was impaired in both the DGKα^+/+^ and DGKα^−/−^ mice.

### The effects of VtE on some markers of DN

**Figure 2:**
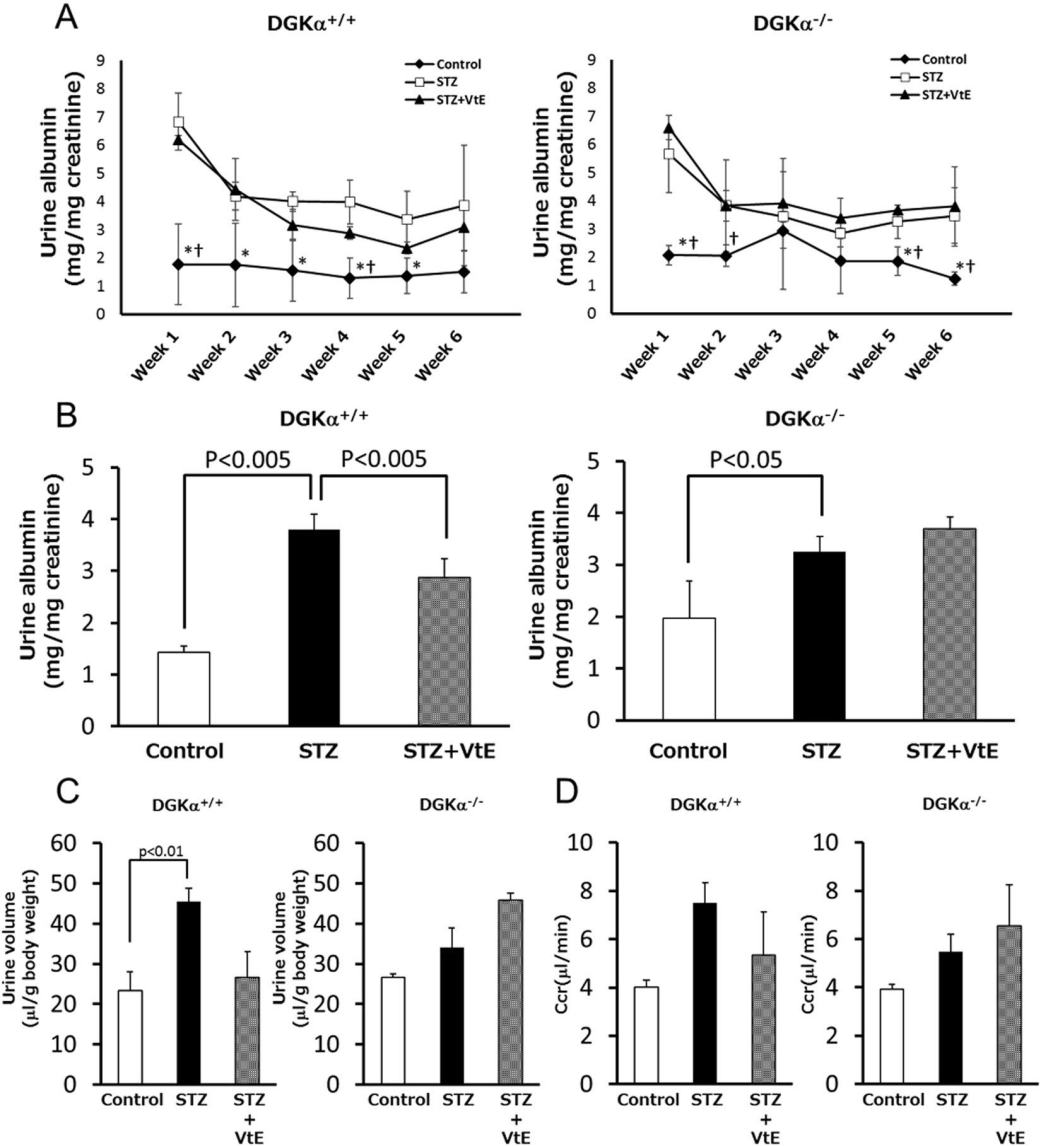
Indicators of diabetic nephropathy. (**A**) Urine albumin levels were measured in each group of mice from weeks 1 to 6. (**B**) The mean amount of urine albumin from weeks 3 to 6. Urine albumin levels were normalized to urine creatinine levels. (**C**) Urine volume was measured for each group of mice using metabolic cages. The bar graphs show data for urine volume at 5 weeks. (**D**) Creatinine clearance (CCr) of mice at 1 week was calculated from the concentrations of urine and plasma creatinine. *P < 0.05 vs. STZ; ^†^P < 0.05 vs. STZ + VtE.

It is well known that urine volume and the amount of urine albumin increase in patients with DN. Creatinine clearance (CCr) is also known to increase at an early stage of DN. Therefore, to investigate whether VtE ameliorates DN *in vivo*, we measured these markers of DN. As shown in Fig. 2A, the urine albumin levels in STZ-treated DGKα^+/+^ and DGKα^−/−^ mice were significantly increased at 1 week after STZ administration, and there was no significant difference in the amount of urine albumin in between the DGKα^+/+^ and DGKα^−/−^ mice until 2 weeks. However, the condition was improved by VtE treatment at 3 weeks in the DGKα^+/+^ mice but not in the DGKα^−/−^ mice, and this improvement lasted until 6 weeks (Fig. 2A). Indeed, the average urine albumin level during weeks 3 to 6 was significantly reduced by VtE treatment in the DGKα^+/+^ mice but not in DGKα^−/−^ mice (Fig. 2B). In addition, the increase in urine volume was also improved by VtE treatment in the DGKα^+/+^ mice but not in the DGKα^−/−^ mice (Fig. 2C).

The DGKα^+/+^ mice showed a tendency towards improved CCr following VtE treatment, but this tendency was not observed in the DGKα^−/−^ mice (Fig. 2D). These results clearly indicate that VtE treatment can ameliorate DN in mice and that DGKα is involved in the VtE-induced amelioration of DN *in vivo*.

### The effects of VtE on the STZ-induced changes in podocyte morphology and podocyte loss

**Figure 3:**
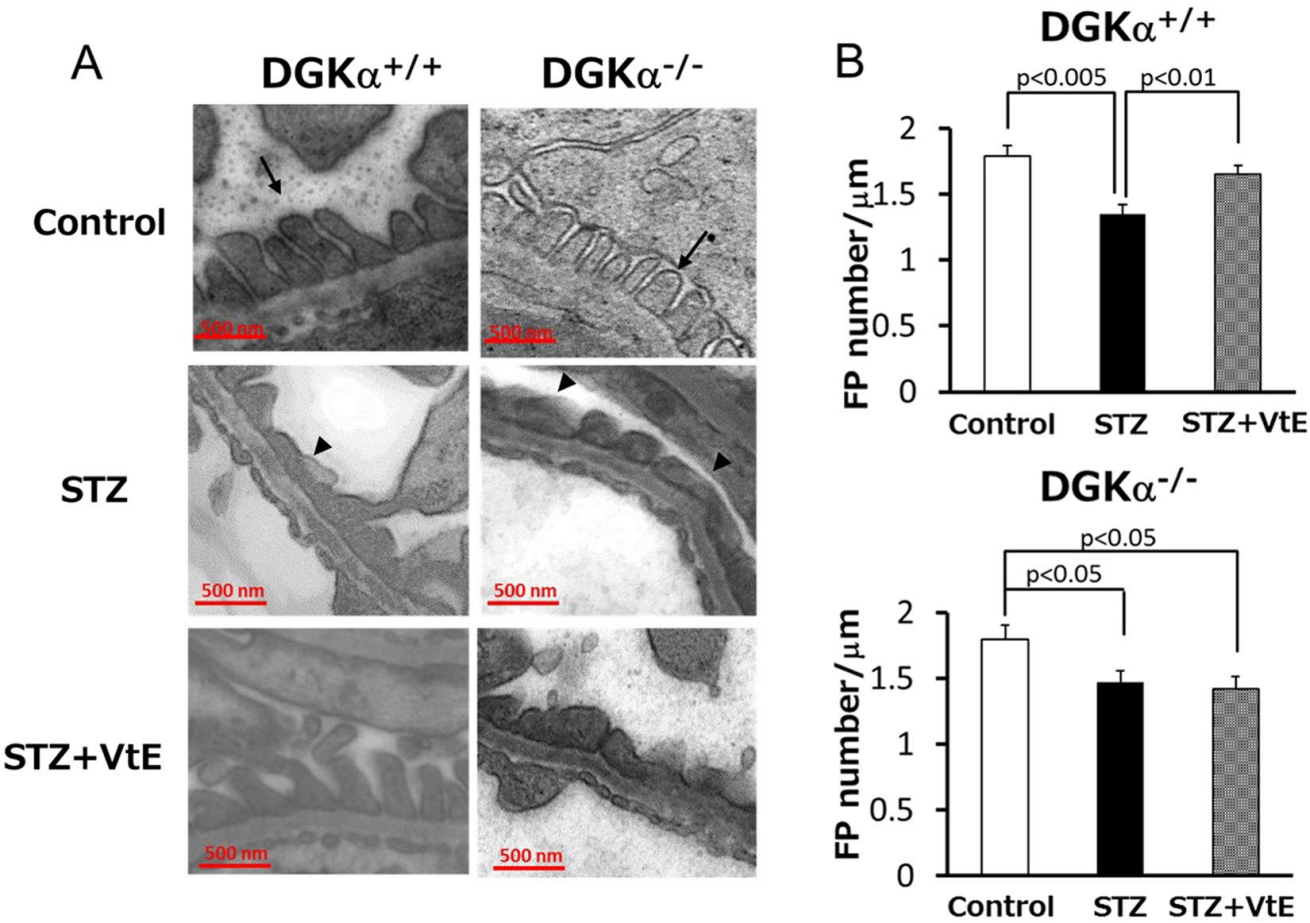
Transmission electron microscopy of podocyte morphology. (**A**) Transmission electron microscopy (TEM) images of glomeruli from mice at 6 weeks after STZ administration. The arrow points to a normal FP, and the arrowhead points to a broadened FP. (**B**) The number of FPs in the TEM images was counted, and the number was normalized to the GBM length.

Glomerular epithelial cells, known as podocytes, extend membrane swellings called foot processes (FPs; indicated by the arrows in Fig. 3A) under normal conditions, and the FPs are interdigitated with each other to form a slit membrane structure that functions as a filtration barrier on the glomerular basement membrane (GBM)^17^. It is well known that DN induces a collapse of podocyte morphology and podocyte loss^18^. Our previous research showed that DGKα is expressed in podocytes by western blotting of cultured podocyte and immunofluorescent staining of mice kidney^19^. Therefore, to investigate the mechanisms underlying the VtE-induced amelioration of DN, we evaluated podocyte morphology by transmission electron microscopy. There was no difference in slit membrane structure between the DGKα^+/+^ and DGKα^−/−^ mice under normal conditions (upper panels of Fig. 3A and Supplementary Figure S1). Following STZ treatment, the morphology of FPs became broad (indicated by the arrowheads in Fig. 3A), and a collapse of the slit membrane structure was observed in both DGKα^+/+^ and DGKα^−/−^ mice (middle panels of Fig. 3A and Supplementary Figure S1). The collapse of podocyte morphology was improved by VtE treatment in the DGKα^+/+^ mice but not in the DGKα^−/−^ mice (lower panels of Fig. 3A and Supplementary Figure S1). As shown in Fig. 3A, FPs were wider in mice with DN than they were control mice; thus, to evaluate the collapse of podocyte morphology, we counted the number of FPs, and the number was normalised to the length of the GBM. The number of FPs was significantly decreased by STZ treatment in both the DGKα^+/+^ and DGKα^−/−^ mice. Interestingly, VtE treatment prevented the STZ-induced decrease in the number of FPs in the DGKα^+/+^ mice but not in the DGKα^−/−^ mice (Fig. 3B).

**Figure 4:**
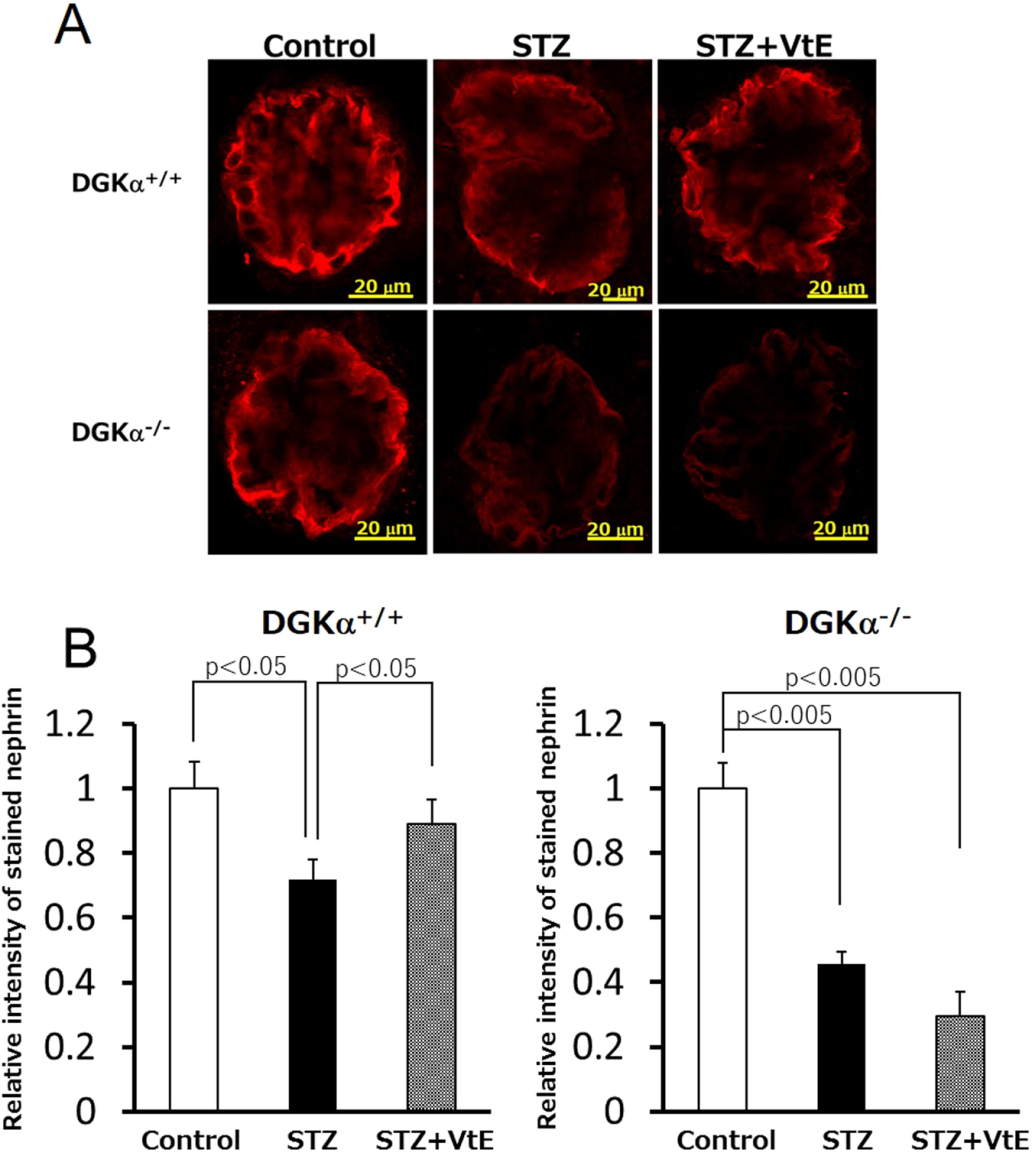
Evaluation of podocyte loss by immunofluorescent staining of nephrin. The kidneys of mice were removed at 6 weeks after STZ administration and were sectioned. We performed immunofluorescent staining of these sections using an anti-nephrin antibody. The nephrin staining (red) was observed using confocal laser microscopy (**A**), and the intensity of stained nephrin was analysed Image J (**B**).

Finally, to evaluate podocyte loss, we performed immunofluorescent staining (IF) of nephrin, which is a marker of podocytes, in kidney glomeruli. There was no difference in the staining pattern of nephrin between the control groups of DGKα^+/+^ and DGKα^−/−^ mice (left panels of Fig. 4A). Compared with the observations from the control groups, the nephrin staining was relatively weaker and the stained area was decreased by STZ treatment in both the DGKα^+/+^ and DGKα^−/−^ mice (middle panels of Fig. 4A). VtE treatment clearly enhanced the nephrin staining pattern in the DGKα^+/+^ mice but not in the DGKα^−/−^ mice (right panels of Fig. 4A), indicating that VtE prevented podocyte loss in the DGKα^+/+^ mice but not in the DGKα^−/−^ mice. Indeed, the fluorescence intensity of stained nephrin was significantly decreased by STZ treatment in both the DGKα^+/+^ and DGKα^−/−^ mice, and VtE treatment significantly prevent the decrease of the intensity only in the DGKα^+/+^ mice (Fig. 4B). These results indicated that VtE ameliorated DN by preventing damage to normal podocyte morphology and preventing the loss of podocytes through DGKα.

## Discussion

In this study, we demonstrated for the first time that VtE treatment can ameliorate DN in mice and, through experiments in DGKα^−/−^ mice, we showed that DGKα has an important role in the VtE-induced amelioration of DN. In addition, we revealed that VtE treatment prevented damage to normal podocyte morphology and prevented the loss of podocytes in DGKα^+/+^ mice but not in DGKα^−/−^ mice. These results suggest that DGKα is involved in maintaining normal podocyte morphology and preventing podocyte loss.

We previously reported that DGKα is expressed in podocytes and translocates to the plasma membrane^19^. Podocytes form a slit membrane structure that functions as a filtration barrier in glomeruli by extending FPs^17^. FPs adhere to neighbouring FPs by slit diaphragm (SD) which is domain of FPs^20^. It is known that nephrin is present in the SD and that it plays a pivotal role in adhesion between FPs^21–23^. Furthermore, podocytes are attached to the GBM via the basal membrane domain (BMD) of FPs^20^. In the BMD, α3β1 integrin facilitates the adhesion of the FPs and the GBM^24, 25^. Namely, nephrin and α3β1 integrin maintain the structure of the slit membrane. Interestingly, it has been reported that DGKα interacts with β1 integrin^26, 27^ therefore, there is possibility that DGKα recruits β1 integrin to the BMD and enhance adhesion of GBM and podocyte to contribute to prevention of podocyte loss. Moreover, PKCα mediates nephrin endocytosis in podocytes^28, 29^. The fact suggests that normalizing of PKC activation contributes to maintaining podocyte morphology. In short, we hypothesise that DGKα maintains podocyte adhesion and morphology by interacting with β1 integrin on the GBM and regulating nephrin in the SD.

It is well known that transforming growth factor-β (TGF-β) and vascular endothelial growth factor (VEGF) are involved in aggravation of kidney disease^30, 31^. PKCα and PKCβII regulate signalling of TGF-β and expression of VEGF in podocyte^32, 33^. As described above, DGKα can attenuate PKC activity by converting DG into PA. Therefore, there is possibility that DGKα attenuate the PKC activity and regulate VEGF and TGF-β to contribute the amelioration of DN. Indeed, STZ treatment induced increase of PKCβII phosphorylation in the kidney, and VtE treatment significantly suppressed the increase of PKCβII phosphorylation in DGKα^+/+^ mice (Supplementary Figure S2).

Before the experiments, we expected that DGKα^−/−^ mice would have an increased severity of DN. Indeed, podocyte loss in DGKα^−/−^ showed an increased severity by STZ treatment compared with DGKα^+/+^ mice, and urine albumin amount of control group of DGKα^−/−^ mice was slightly higher than that of DGKα^+/+^ mice although it was not significant. However, almost all symptoms of DN were not exacerbated in the STZ-treated DGKα^−/−^ mice against our prediction, for example, urine volume (DGKα^+/+^: 45.6 μl/g, DGKα^−/−^: 34.1 μl/g) and CCr (DGKα^+/+^: 7.50 μl/min, DGKα^−/−^: 5.48 μl/min) suggesting that ease of injury by hyperglycaemia easily occurs in DGKα^−/−^ mice compared with the DGKα^+/+^ mice. In other words, abolishment of improvement effect of VtE on markers of DN was not caused by difference in the ease of injury by hyperglycaemia. The reason that the DGKα^−/−^ mice did not show severe symptoms of DN may be related to the expression of other DGK isoforms in the kidney, which may compensate for the loss of DGKα function. Indeed, it was previously reported that DGKβ, DGKγ, DGKε, and DGK ζ are also expressed in the kidney^15^. Among them, not only DGKα but also DGKε might be important to maintaining glomerular function. Recently, some groups reported that mutation in the DGKε gene caused kidney disiase^34, 35^. Moreover, it was also reported that the DGKε deficient mice showed multiple glomerular failures and that damage to morphology of normal podocyte^36^. Indeed, DGKε deficient mice have an increased severity of albuminuria by nephrotoxic serum which induces nephritis^36^.

The oxidative stress is known as one of causes of DN^37^ and VtE is well known as an antioxidant. It was reported that the expression of heme oxygenase-1 (HO-1) which is an antioxidant enzyme increases in glomerulus of diabetic rats and the expression of HO-1 was normalized by VtE^38^. In our study, the expression of HO-1 was suppressed by VtE treatment compared with STZ-induced diabetic mice in DGKα^+/+^ mice (Supplementary Figure S3). This fact indicates that VtE acts as not only an activator of DGKα but also an antioxidant. In addition to VtE, our previous research revealed that an antioxidant green tea polyphenol epigallocatechin-gallate (EGCg) also activates DGKα and showed that EGCg induces the translocation of DGKα to the plasma membrane in cultured mouse podocytes^19^. Recently, C. M. Borges *et al*. reported that EGCg ameliorated albuminuria in DN in human clinical trials and showed that EGCg prevented podocyte apoptosis^39^. We predict that DGKα is involved in the amelioration of DN in humans through mechanisms similar to those observed in this study. Thus, DGKα may be an attractive therapeutic target for DN. Therefore, we are conducting research into the function of DGKα in human podocytes.

In conclusion, this study shows that VtE ameliorated DN in mice and that DGKα is involved in the VtE-induced amelioration of DN *in vivo* by regulating podocyte morphology and by preventing the loss of podocytes.

## Methods

### Materials

Streptozocin (STZ) was purchased from SIGMA-Aldrich (St. Louis, MO, USA). DL -α-tocopherol (VtE) was purchased from Wako (Kobe, Japan). Glu-testSensor and Glu-testEvery were purchased from Sanwa Kagaku Kenkyuusho (Nagoya, Japan).

### Mice

All animal study was approved by the Kobe University Institutional Animal Care and Use Committee (Permission number: 25-07-03) and carried out according to the Kobe University Animal Experimentation Regulations. Wild-type C57BL/6 (DGKα^+/+^) mice were purchased from Japan SLC, Inc. (Shizuoka, Japan). DGKα-knockout (DGKα^−/−^) C57BL/6 mice were a gift from Dr. Topham (University of Utah). All mice received a normal diet and had free access to water. The mice were bred under a 12-hour light-dark cycle, and the temperature was maintained at approximately 18–26 °C. To induce diabetes, six-week-old male DGKα^+/+^ and DGKα^−/−^ mice were intraperitoneally (i.p.) administered STZ (50 mg/kg) in 20 mM citrate buffer once a day for 5 consecutive days. For the control group, the same volume of vehicle was administered (i.p.). For the VtE-treated group, VtE (40 mg/kg) was administered (i.p.) to mice with STZ-induced diabetes (both DGKα^+/+^ and DGKα^−/−^ mice) every other day after the final STZ administration. Fasting blood glucose levels and body weight were measured every week after the final STZ administration.

### Urine and plasma analysis

Under fasting conditions, urine was collected from mice for 8 h (9:30 am~5:30 pm) using metabolic cages. The volume of collected urine was measured, and the urine was centrifuged at 3,000 rpm for 10 min. The albumin and creatinine analyses of the urine supernatant were conducted by Oriental Yeast Co., Ltd. (Tokyo, Japan). After urine collection, blood was collected from the tails of mice into a microtube containing Novoheparin (Mochida Pharmaceutical Co., Ltd., Tokyo, Japan). The collected blood was centrifuged at 3,000 rpm for 10 min, and the supernatant was used for plasma creatinine analysis. The plasma creatinine concentration was measured by LC-MS/MS^40^.

### Immunofluorescent staining of nephrin in kidney glomeruli (evaluation of podocyte loss)

At the end of the experiment, mice from each group were sacrificed and perfused with 0.9% NaCl. The kidneys were removed and embedded in O.C.T. compound. After freezing at −30 °C, the kidney samples were sliced into 20 μm sections using a cryostat (Leica CM1850). The sections were fixed in acetone, and immunofluorescence staining was carried out using a guinea-pig anti-nephrin antibody (PROGEN Biotechnik, Heidelberg, Germany) as the primary antibody and an Alexa Fluor-conjugated secondary antibody. Finally, the fluorescence signal was observed using confocal microscopy.

### Transmission electron microscopy (evaluation of podocyte effacement)

Six weeks after the final STZ administration, mice were sacrificed and perfused with a fixing solution (containing 4% PFA and 0.2% glutaraldehyde in 0.1 M phosphate buffer) via left ventricular puncture. The kidneys were removed and cut into 2 mm cubes. These cubes were fixed in the fixing solution for 6 h at 4 °C and the cubes incubates in 0.1 M phosphate buffer containing 4% OsO_4_ for 16 h at 4 °C. After the tissue was fixed, the cubes were dehydrated with ethanol and embedded in resin. Embedded cubes were sliced using an ultra-microtome (Leica) into sections with a thickness of 100 nm, and these sections were observed using transmission electron microscopy. To evaluate podocyte effacement, we counted the number of foot processes (FPs) of the podocytes, and the number was normalised to the length of the glomerular basement membrane (GBM).

### Statistical Analyses

Student’s t-tests were used as appropriate to test statistical significance and p value of less than 0.05 was considered to be significant.

## Electronic supplementary material

Supplementary information
