# Supplementary material for: Diacylglycerol Kinase alpha is Involved in the Vitamin E-Induced Amelioration of Diabetic Nephropathy in Mice
Source: Sci Rep. 2017 Jun 1;7:2597. doi: 10.1038/s41598-017-02354-3 (PMC5453949; doi:10.1038/s41598-017-02354-3)
Supplement: Supplementary file 1 — Supplementary information [file 41598_2017_2354_MOESM1_ESM.pdf]

Supplementary Information

Diacylglycerol Kinase alpha is Involved in the Vitamin E-Induced Amelioration of Diabetic Nephropathy in Mice

Daiki Hayashi[1], Keiko Yagi[2], Chihong Song[3], Shuji Ueda[1], Minoru Yamanoue[1], Matthew Topham[4], Toshinobu Suzaki[3], Naoaki Saito[5], Noriaki Emoto[2], and Yasuhito Shirai[1*]

1. Department of Applied Chemistry in Bioscience, Graduate School of Agricultural Science, Faculty of Agriculture, Kobe University, Kobe, Japan
2. Department of Clinical Pharmacy, Kobe Pharmaceutical University, Kobe, Japan
3. Department of Biology, Graduate School of Science, Faculty of Science, Kobe University, Kobe, Japan
4. Huntsman Cancer Institute, University of Utah, Salt Lake City, UT, USA
5. Laboratory of Molecular Pharmacology, Biosignal Research Center, Kobe University, Kobe, Japan

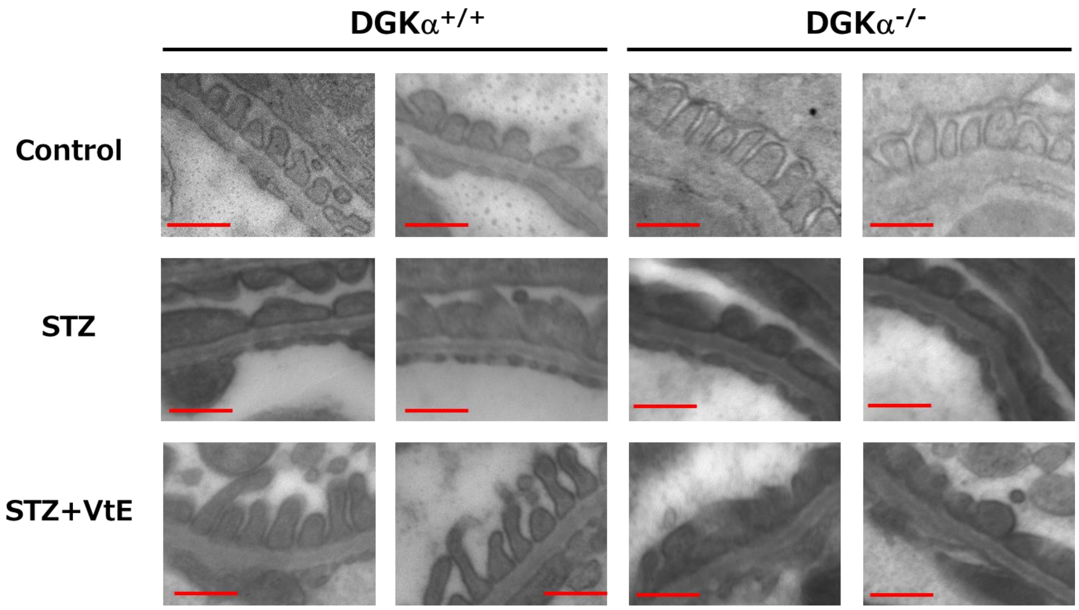

Supplementary Figure S1: Other images of Fig. 3A

Transmission electron microscopy (TEM) images of glomeruli from mice at 6 weeks after STZ administration. Red bars indicate 500 nm.

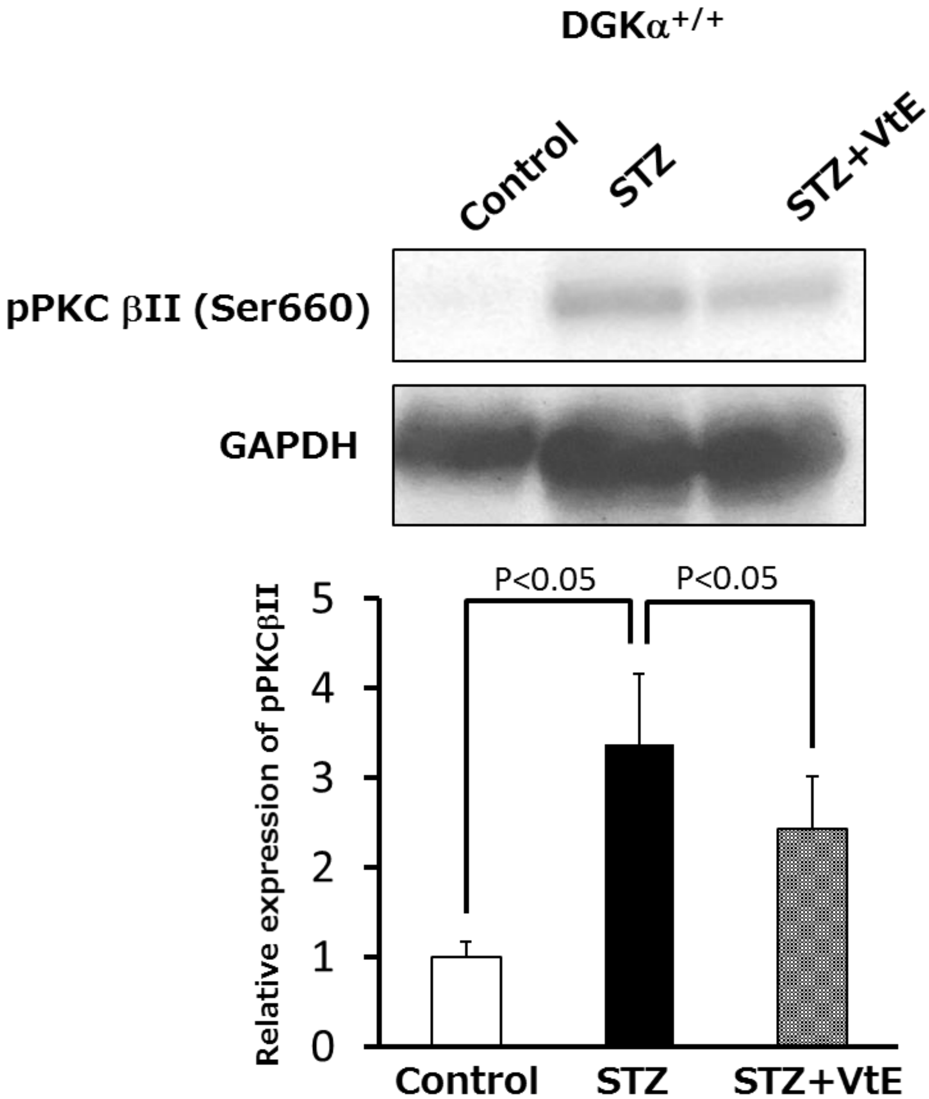

Supplementary Figure S2: VtE suppress phosphorylation of PKCβII

The expression of phosphorylated PKCβII (Ser660) in kidney from DGKα$^{+/+}$ mice at week 6 after STZ administration were measured by western blotting.

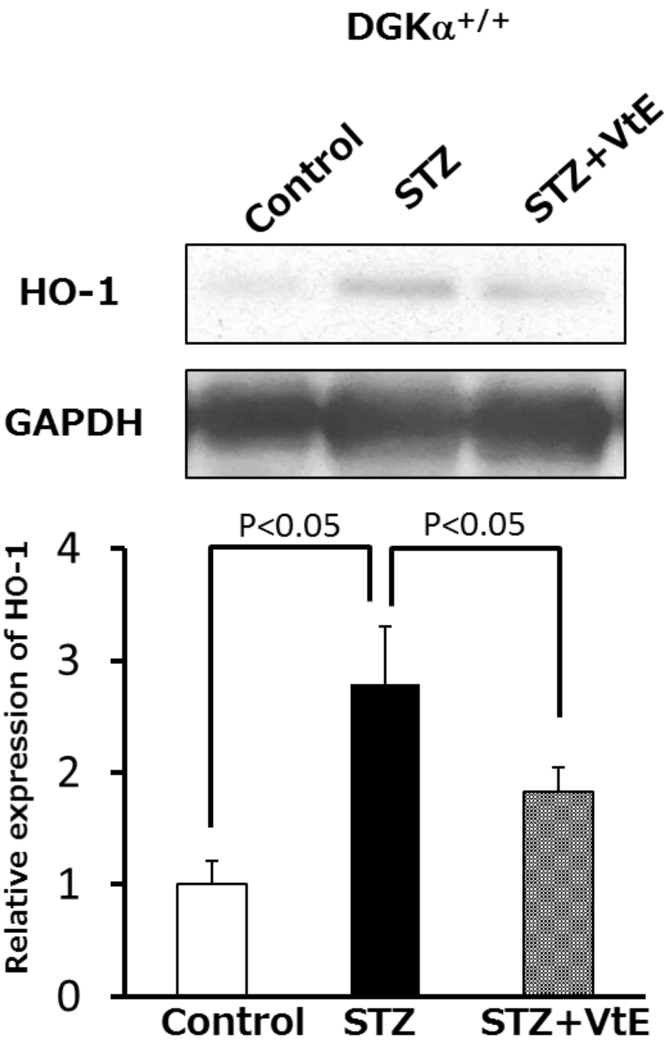

Supplementary Figure S3: VtE suppress the expression of HO-1

HO-1 expression in kidney from DGK$\alpha^{+/+}$ mice at week 6 after STZ administration were measured by western blotting.
